# Influence of Baseline Cardiovascular Comorbidities on Mortality after Androgen Deprivation Therapy for Metastatic Prostate Cancer

**DOI:** 10.3390/cancers12010189

**Published:** 2020-01-12

**Authors:** Szu-Yuan Wu, Su-Chen Fang, Olivia Rachel Hwang, Hung-Jen Shih, Yu-Hsuan Joni Shao

**Affiliations:** 1 Department of Food Nutrition and Health Biotechnology, College of Medical and Health Science, Asia University, Taichung 413, Taiwan; szuyuanwu5399@gmail.com; 2 Division of Radiation Oncology, Department of Medicine, Lo-Hsu Medical Foundation, Lotung Poh-Ai Hospital, Yilan 265, Taiwan; 3 Big Data Center, Lo-Hsu Medical Foundation, Lotung Poh-Ai Hospital, Yilan 265, Taiwan; 4 Department of Healthcare Administration, College of Medical and Health Science, Asia University, Taichung 413, Taiwan; 5 Department of Nursing, Mackay Medical College, New Taipei City 252, Taiwan; scfang002@gmail.com; 6 Department, Graduate Institute of Biomedical Informatics, Taipei Medical University, Taipei 110, Taiwan; weiwei2392@gmail.com; 7 Department of Urology, Wan Fang Hospital, Taipei Medical University, Taipei 106, Taiwan; jasta1206@gmail.com; 8 Department of Urology, School of Medicine, Taipei Medical University, Taipei 110, Taiwan; 9 Clinical Big Data Research Center, Taipei Medical University Hospital, Taipei 110, Taiwan

**Keywords:** prostate cancer, androgen deprivation therapy, survival, cardiovascular diseases, age

## Abstract

Few studies have assessed the benefits of androgen deprivation therapy (ADT) in men with metastatic prostate cancer (PC; mPC) at an old age or with major cardiovascular conditions. A retrospective cohort consisted of 3835 men with newly diagnosed mPC from the Taiwan Cancer Registry of 2008–2014. Among them, 2692 patients received only ADT in the first year after the cancer diagnosis, and 1143 patients were on watchful waiting. The inverse probability of treatment-weighted Cox model was used to estimate the effects of ADT on all-cause mortality and PC-specific mortality according to age, and the status of congestive heart failure (CHF), coronary arterial diseases (CADs), and stroke at the baseline. After a median follow-up of 2.65 years, 1650 men had died. ADT was associated with a 17–22% risk reduction in all-cause and PC-specific mortality in men without stroke, CAD, or CHF in the 65–79-year group. The survival benefit diminished in men with any of these preexisting conditions. In contrast, ADT was not found to be associated with any survival benefit in the ≥80-year group, even though they did not present with any major cardiovascular disease at the baseline. Patients who had CHF, CAD, or stroke at the baseline did not show a survival benefit following ADT in any of the age groups. Men who have preexisting major cardiovascular diseases or are ≥80 years do not demonstrate a survival benefit from ADT for mPC. The risk–benefit ratio should be considered when using ADT for mPC in older men especially those with major cardiovascular comorbidities.

## 1. Introduction

More than 5000 men are diagnosed with prostate cancer (PC) in Taiwan every year and almost half of these patients diagnosed at an advanced stage. The incidence increased rapidly and it became the top 6 cancer in Taiwan [1]. Androgen deprivation therapy (ADT) leading the chemical castration suppresses testosterone production and provides remission of PC. ADT is the first line treatment for metastatic PC (mPC) and it is the most common treatment for PC [2]. However, ADT has side effects, such as sexual dysfunction and osteoporosis [3]. Evidence also found increased risks in developing insulin resistance, hypertension, dyslipidemia, change in body composition, and obesity following ADT [4,5,6,7,8]. ADT-induced metabolic syndrome related complications might be responsible for cardiovascular events and increase all-cause mortality [9]. 

Age and comorbidity are independent prognostic factors for many cancers and have an impact on treatment choice [10,11]. This is particularly true for PC because the PC is usually a disease in elderly men. Currently, the National Comprehensive Cancer Network (NCCN) guidelines recommend a 5-year life expectancy cut-off to determine the need for further interventions in men diagnosed with PC [12]. It is speculated that the treatment benefits may easily be outweighed in older patients or those with comorbid conditions who have a limited life expectancy. However, few studies assessed the benefits of ADT in older men, especially in those with cardiovascular comorbidities and mPC. 

Watchful waiting is reported as an acceptable alternative for men with a short life expectancy based on age and/or substantial comorbidity [13,14,15]. A study evaluated outcomes in 3183 men who were included in the Prostate Cancer Outcomes Study (PCOS). Results found that older men with multiple major comorbid conditions are at a high risk for other-cause mortality within 10 years of diagnosis and might consider conservative management [16]. As noted above, the 2019 consensus-based guidelines from the EAU/ESTRO/SIOG recommend the use of ePrognosis in the setting of localized PC to estimate non-cancer-specific life expectancy and aid in decision making [17]. However, the benefit of ADT use and elderly mPC patients with major cardiovascular diseases was not considered separately. Cardiovascular diseases are very common in elderly men [18,19,20]. The estimated lifetime risk for men aged 70 years of developing coronary heart diseases is 34.9% [21]. Similar findings were reported in a meta-analysis of 18 cohorts, including over 250,000 men and women [22]. The three leading causes of death among cardiovascular comorbidities (major cardiovascular comorbidities) are congestive heart failure (CHF), coronary artery disease (CAD), and stroke [23,24,25,26]. Although PC mostly affects men in their old age, many of them have preexisting cardiovascular conditions when they are diagnosed with cancer. Previous studies showed that patients with a recent history of major cardiovascular diseases are more likely to have adverse cardiovascular events within the first 6 months of gonadotropin-releasing hormone (GnRH) agonist treatment [27]. In men with high-risk localized PC, patients who had CHF or a myocardial infarction had higher all-cause mortality after neoadjuvant ADT than those who only received radiation [28].

For several decades, men with mPC had few treatment options besides ADT as their primary approach. Studies examining the benefits of ADT among men with major cardiovascular comorbidities are scarce. Very few studies have attempted to identify specific comorbidities that predispose men to die without realizing a benefit from ADT. In this study, we examined the all-cause mortality and PC-specific mortality (PCSM) associated with ADT among men with mPC, and considered preexisting cardiovascular comorbidities at the baseline. We also evaluated the influence of cardiovascular comorbidities on mortality in different age groups to provide patients and clinicians with better estimates of the benefits of ADT.

## 2. Patients and Methods

### 2.1. Study Population

A population-based cohort study was conducted using Taiwan National Health Insurance (NHI) Research Data (NHIRD) linked to the Taiwan Cancer Registry (TCR). The TCR was established in 1979 for cancer surveillance, and includes 97% of all cancer cases in Taiwan [29]. NHIRD is obtained from the National Health Insurance program with more than 25.7 million beneficiaries. It covers more than 99% of the population in Taiwan. The NHIRD includes medical claims data of disease diagnoses, procedures, drug prescriptions, demographics, and enrollment profiles of all NHI beneficiaries [30]. The NHIRD and TCR are linked by encrypted patient identifiers. NHIRD are additionally linked to the Death Registry to ascertain the vital status and cause of death of each patient. Data quality of TCR was examined by previous studies showing a good level of accuracy [29].

Our cohort included 28,983 PC patients in 2008–2014 from TCR using the ICD-O-3 site-specific code (C61.9). We excluded patients with missing data of age, date of the cancer diagnosis, or cancer stage (*n* = 2801), and those who had had another cancer diagnosis before PC (*n* = 1934). We also excluded those who died within 1 year after the cancer diagnosis (*n* = 1630) to ensure all patients having equal opportunities to receive treatment and enough time to demonstrate the treatment effect. We further identified patients who were diagnosed with localized or regional diseases (T1–T4, N0M0) (*n* = 14,595) and lymph nodes or distant metastasis (N1M0, Any N M1+) (*n* = 8023) by the American Joint Committee on Cancer (AJCC) staging system [31]. TNM staging has been incorporated into a staging system that was developed by the AJCC in collaboration with the Union for International Cancer Control (UICC). This system relies upon assessments of the primary tumor (T), regional lymph nodes (N), and distant metastatic sites (M) [32]. Men with clinical evidence of lymph node involvement (cN1) are classified as having stage IV disease, regardless of the extent of their primary tumor [32]. Patients with metastasis considered as stage IV are subdivided into those with non-regional lymph node involvement (M1a), bone metastases (M1b), or other metastatic sites with or without bone involvement (M1c) [32]. Among patients with N1 or M1+ diseases, we further excluded those aged under 65 years (*n* = 1132) because early onset PC are suggested a distinct phenotype, which is usually aggressive [33]. We also excluded those who had received surgery, radiation, or local treatment in the first year after their cancer diagnosis (*n* = 3056). Surgery and radiotherapy have been considered as definitive therapy with curative-intent [12]. We only excluded definitive RT to primary cancer sites, but palliative RT for symptoms or signs relief were allowed. After applying the exclusion criteria, a total of 3835 PC patients who had found cancer cells in either lymph nodes near the prostate (N1) or cancer has spread to distant sites outside of pelvis (M1) were included.

### 2.2. ADT

We defined the ADT group as those who received at least three months of goserelin, leuprolide, and triptorelin (ATC codes: L02AE03, L02AE02, L02AE04), or combined with antiandrogens (bicalutamide, cyproterone, and flutamide; ATC codes: L02BB03, G03HA01, L02BB01), estrogens (ATC code: L02AA), and ketoconazole (ATC code: J02AB02). Advanced PC patients are suggested to receive continuous ADT in Taiwan [2]. Therefore, no intermittent treatment was included in this study. In total, we found 2692 patients who received only ADT in the first year after their cancer diagnosis and 1143 patients who were untreated. The effect of ADT was defined as Yes/No and modeled in the analysis.

### 2.3. Study Covariates

All variables known a priori to be strongly, or at least moderately, associated with the primary outcomes were selected. The age at cancer diagnosis, previous and coexisting medical conditions, cancer grade, cancer stage, year of the cancer diagnosis, and secondary treatment in the study were included as covariates. The study population was divided into the following age cohorts, <80 and ≥80 years at the time of the cancer diagnosis. Clinical stage was categorized into N1M0 or M1 using the 7th edition AJCC [31,34]. The TCR describes cancer grades as “well”, “moderately”, and “poorly differentiated” based on respective Gleason scores of 2–6, 7, and 8–10.

Comorbidities at the baseline were estimated by coexisting conditions which had occurred during 1 year prior to the PC diagnosis. We individually examined the effects of diabetes (International Classification of Disease, Ninth Revision, Clinical Modification (ICD-9-CM): 250), hypertension (ICD-9-CM: 401–405), stroke (ICD-9-CM: 433–436, 453), CHF (ICD-9-CM: 428), and CADs (ICD-9-CM: 410–414, 440–448), because these are common comorbidities among elderly men and were found to impact patient survival [35,36]. Furthermore, we searched the NHIRD claims records for chemotherapy, new-generation antiandrogens, enzalutamide, and abiraterone from 365 days after the cancer diagnosis to define secondary treatment in these patients.

### 2.4. Outcome Variables

The primary outcomes were all-cause, PC-specific, and cardiovascular (CV)-specific mortality. The occurrence of all-cause, PC-specific, and CV deaths was determined from the death registry cause of death data from the PC diagnosis until death or the end of the study (31 December 2016). Cause-specific deaths were defined by ICD-10 using C61 for PC deaths and I00-I99 for CV deaths. The accuracy of cause-of-death was varied in a previous study and the cancer causes reached a high level of accuracy [37].

### 2.5. Statistical Analysis

Descriptive analyses and statistics were performed to compare baseline characteristics among patients receiving ADT and those who were untreated. A *t*-test was adapted for mean age, and a Chi-squared test for categorical variables. A Q-Q plot approach was used to graphically test the normality of age (Supplemental Figure S1). We used the inverse probability of treatment-weighted (IPTW) Cox regression models to assess the effects of variables on the associations of interest [38]. IPTW using the propensity score to adjust the imbalance in baseline characteristics between the ADT and untreated groups; it allows us to obtain unbiased estimates of average treatment effects considering patients’ age, cancer stage, cancer grade and year of cancer diagnosis [38]. IPTW Cox models were used to compare overall, PC-, and CV-specific survival of men receiving and those not receiving ADT. In these models, we adjusted for the confounding effects from secondary treatment, and comorbidities at the baseline. The Fine and Gray method was adapted to estimate the hazard of PC- and CV-specific survival considering competing risks from other causes of death [39]. The hazard ratio (HR) and 95% confidence interval (CI) estimating the risk of death associated with patients receiving ADT treatment compared those without treatment were calculated. 

First, we examined the mortality risk associated with ADT, stroke, CHF, and CAD. We further examined the mortality risk in men receiving ADT compared with those with by patients’ age and status with or without stroke, CHF, and CADs at the baseline. In addition, survival curves and associated HRs of men receiving ADT and those without were generated in patient subgroup by age (<80, ≥80) and by the status of cardiovascular comorbid conditions at baseline (without any conditions, with at least one condition). 

All statistical analyses were conducted using SAS ver. 9.4 (SAS Institute, Cary, NC, USA). Relationships were considered statistically significant at *p*-values of < 0.05. This study was reviewed and approved by the Institutional Review Board of Taipei Medical University (TMU-JIRB no. 201502042), and the need to obtain informed consent was waived. NHIRD requires that investigators may not publish or present findings in which the number of cases in a cell is less than three. This policy is to eliminate the potential for re-identification of persons with specific disease or condition.

## 3. Results

### 3.1. Baseline Characteristics

This study cohort consisted of 3835 men aged ≥65 years with advanced PC diagnosed between 2008 and 2014. Among them, 2692 (70.1%) men had received only ADT as their primary treatment in the first year, and 1143 (29.9%) men had received watchful waiting (Table 1). The median follow-up time for overall survival was 2.65 years (interquartile range (IQR) = 1.56). The mean follow-up time for overall survival was 3.12 years (standard deviation (SD) = 1.70). Patients receiving ADT and those without had similar ages (*p* = 0.62). Men receiving ADT had a lower percentage of distant metastatic disease (*p* < 0.0001), fewer stroke (*p* = 0.0191), and fewer CHF (*p* = 0.0272) than men without treatment. However, they had higher cancer grades at the time of the cancer diagnosis than their counterparts (*p* < 0.0001). We also presented the baseline characteristics by age group of <80 and ≥80 years (Supplemental Table S1). No significant differences in major cardiovascular comorbidities were observed between patients receiving ADT and those without in both age groups.

### 3.2. Mortality Associated with Baseline Cardiovascular Comorbidities and ADT

During the follow-up, 1650 deaths from all causes, 1161 deaths from PC, and 381 deaths from major cardiovascular diseases observed in the study cohort (Table 1). Table 2 shows the results of three models estimating the risk of death associated with ADT, CHF, stroke, CADs and other variables for all-cause, and PC- and CV-death, respectively. Overall, ADT was associated with improved all-cause and PC-specific mortality, but not associated with CV mortality in our patients. In these models, we found that patients with CHF have a higher risk of all-cause, PC-specific and CV mortality comparing with those without CHF at baseline while adjusting for other variables. Patients who had CHF at the baseline demonstrated a 17–59% increased risk of all-cause, PC-specific, and CV mortality after adjusting for ADT and the status of stroke and coronary artery disease at the baseline, when compared with those with CHF at baseline.

### 3.3. Impacts of Cardiovascular Conditions at the Baseline on Mortality Following ADT

We examined the risk of mortality associated with ADT by age and the status of major cardiovascular diseases present at the baseline (Table 3). Among patients aged <80 years, ADT was associated with a significant reduction in all-cause and PC mortality in patients who did not have CHF, stroke, or CAD at the baseline. However, the benefit of ADT in reducing all-cause and PC mortality was not observed in patients who had CHF, stroke, or CAD at the baseline. ADT was associated with an increased risk of CV mortality in patients who presented with major cardiovascular diseases at the baseline, but none of the estimates reached statistical significance. Among patients aged ≥80 years, ADT showed a borderline association with mortality, but none of the estimates was significant.

**Figure 1:**
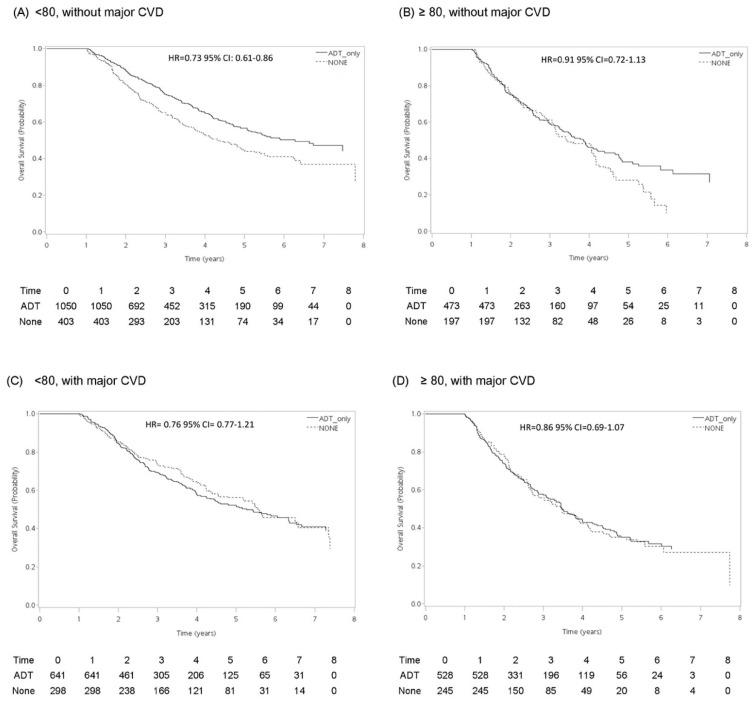
Inversed probability weighted survival estimates for all-cause mortality in men with metastatic prostate cancer receiving androgen deprivation therapy vs. watchful waiting by age group and the status of baseline cardiovascular comorbidities (major cardiovascular diseases): (**A**) <80 years old without major cardiovascular diseases, (**B**) ≥80 years old without major cardiovascular diseases, (**C**) <80 years old with major cardiovascular diseases, and (**D**) ≥80 with major cardiovascular diseases.

Figure 1 presents survival curves associated with ADT in patients with and those without major cardiovascular diseases in different age groups. ADT was associated with a reduced risk (HR = 0.73, 95% CI: 0.61–0.86) in all-cause mortality in men without stroke, CAD, or CHF in the <80-year age group compared to the no-treatment group. However, the survival benefits diminished in men with any of these three pre-existing conditions. In contrast, ADT was not associated with any survival benefit in men aged ≥80 years in either those with or without major cardiovascular diseases at the baseline.

The status of major cardiovascular diseases at baseline was defined as men diagnosed with or without any of the following conditions: congestion heart failure, stroke or artery diseases during 1 year prior to cancer diagnosis. Inverse probability weighted survival estimates adjusting for secondary treatment was calculated. Hazard ratio (HR) and 95% confidence interval (CI) were estimated by Cox regression.

## 4. Discussion

ADT has been the standard of care in men with hormone-sensitive mPC for several decades. Because of the superb disease control offered by ADT in managing mPC, few studies have assessed the benefits of ADT in men with major cardiovascular comorbidities. Currently, National Comprehensive Cancer Network (NCCN) guidelines recommend a 5-year life expectancy cut-off to determine the need for further interventions in men diagnosed with PC [12]. Therefore, it is not surprising that our analysis did not find that ADT use was associated with improved survival in men who were older than 80 years, even in those who were healthy. However, we further discovered that men who presented with CHF, stroke, or CAD at the time of the cancer diagnosis did not demonstrate a survival benefit from ADT in any age group compared to patients who were treated conservatively. ADT is not complication free; therefore, the risk–benefit ratio should be considered in managing mPC in elderly men, especially those with major cardiovascular comorbidities.

Results of studying ADT-induced major cardiovascular diseases in men with PC are so far conclusive [28,40,41,42,43]. However, a previous study reported that neoadjuvant ADT was associated with an increased risk of all-cause mortality among men with CHF or myocardial infraction in high-risk localized PC [28]. Another study that examined a cohort from Sweden reported that patients who had two or more prior cardiovascular comorbidities were more likely to develop fatal or non-fatal cardiovascular events in the first 6 months of hormonal therapy [27]. Cumulative evidence suggests that coexisting cardiovascular conditions do contribute to patient outcomes. In this study, we evaluated impacts of major cardiovascular comorbidities on outcomes of men with mPC following ADT in different age strata. Our results fill an important gap in current practice while NCCN-predicted life expectancy and other life tables are substantially heterogeneous in each comorbidity group, which are too general to adapt to individual patients [44]. Major cardiovascular comorbidities, stroke, CHF, or CADs in elderly patients should be strongly considered in addition to age when deciding on appropriate management for patients with mPC.

Findings from the CHAARTED, LATITUTE, and STAMPEDE trials showed that initiating docetaxel or abiraterone with ADT may increase disease control in men with PC [45,46,47]. This initiated a clinical challenge for choosing the best treatment regimen for patients. Although these new agents are pricy and come with a certain level of side-effects, choosing therapies for patients with mPC requires careful consideration of each patient’s disease characteristics, comorbidities, and possible treatment-related toxicities. Current evidence is lacking to prove the effectiveness of these new agents plus ADT in men of advanced age or with cardiovascular comorbidities. In addition, results showed that the adverse event profiles of docetaxel and abiraterone differed. In the CHAARTED and arm C of the STAMPEDE trial, there were more cardiac-related toxicities found in patients receiving abiraterone compared to a placebo [46,47]. In contrast, neutropenia, febrile neutropenia, fatigue, and sensory neuropathies were the most commonly seen grade 3/4 toxicities in patients receiving docetaxel. New evidence has shown that patients with low-volume metastases did not benefit from ADT plus early docetaxel [48]. Therefore, further investigations should be aimed at evaluating whether docetaxel or abiraterone added to ADT can provide survival benefits for patients with major cardiovascular comorbidities.

The strength of this study was its large sample size of patients with newly diagnosed mPC. In this cohort, 43% of patients died during the study period, and 70% of these deaths were PC-related, which provided us sufficient power to detect mortality outcomes. The clinical significance of this finding is that specific comorbidities presenting at the baseline may predict one’s life expectancy better than age itself. Although results from randomized control trials usually exclude patients with comorbidities, our results provide a better estimation of survival benefits associated with ADT in a real-world population.

This study had some limitations. First, all patients with PC included in this study were of Asian descent, and the corresponding ethnic susceptibility is unclear; therefore, our results should be cautiously extrapolated to non-Asian populations. The prevalence of major cardiovascular diseases in Northeast Asia is relatively lower than that in Caucasians [49]. Impacts of cardiovascular comorbidities on mortality associated with ADT may be greater in Caucasian populations than we observed in this study, as heart disease is the most common cause of death in PC survivors among Caucasian populations [50]. Second, new treatments for metastatic diseases did not become popular in Taiwan until the past few years. Therefore, relatively lower numbers of patients received these treatments in our cohorts. We could not evaluate whether cardiovascular comorbidities influenced the treatment effects in men who received ADT alone with any of these newer treatments. Third, all diagnoses of comorbid conditions were abstracted from medical claims using ICD-9-CM codes. Although we restricted those conditions diagnosed within one year before the cancer diagnosis, the severity of these preexisting conditions might not have been the same in every patient. However, these major cardiovascular conditions that we focused on in this study are all associated with notable mortality especially in the elderly. The magnitude of the influence on the life expectancy would be higher than we observed if we had focused on patients with severe conditions. Last, although we made our best effort to remove potential bias from our study, there is still a possibility of unmeasured confounding due to the retrospective nature.

## 5. Conclusions

This retrospective cohort study identified specific comorbidities that predispose men to high mortality. Men with mPC presenting with a history of CHF, stroke, and CADs at the baseline or who were older than 80 years of age were unlikely to gain survival benefits from ADT. However, robust data provided from future, larger prospective studies are required to validate the results. Till then, the treatment of this specific cohort of patients diagnosed with mPC should be applied according to guidelines.

## Figures and Tables

**Table 1:** Distribution of baseline patient characteristics according to the status of androgen deprivation therapy (ADT) within the first year after a cancer diagnosis.

Variables	ADT (*N* = 2692)		Watchful Waiting (*N* = 1143)		*p*-Value *
	*n*	%	*n*	%	
Mean age at diagnosis, years (SD)	77.05 (6.56)		77.30 (6.70)		0.29 **
Age at diagnosis (years)					0.62
65–74	988	36.7	410	35.9	
75–79	703	26.1	291	25.5	
≥80	1001	37.2	442	38.7	
Cancer stage					<0.0001
N1M0	385	14.3	61	5.34	
Any N M1	2307	85.7	1082	94.66	
Cancer grade					<0.0001
Well differentiated	56	2.08	46	4.02	
Moderately differentiated	388	14.41	125	10.94	
Poorly differentiated	2133	79.23	769	67.28	
Unknown	115	4.27	203	17.76	
Major cardiovascular comorbidities					
Stroke	806	29.94	386	33.77	0.0191
Congestive heart failure (CHF)	512	19.02	253	22.13	0.0272
Coronary artery disease (CAD)	848	31.50	369	32.28	0.6338
Other comorbidities					
Diabetes	1033	38.37	458	40.07	0.3241
Hypertension	2102	78.08	910	79.62	0.2906
Secondary treatment					<0.0001
Chemotherapy	308	11.44	-		
Enzalutamide/Abiraterone	14	0.52	-		
Combination	85	3.16	-		
None	2285	84.88	1143	100	
Calendar Year					<0.0001
2008	240	8.92	182	15.92	
2009	272	10.1	161	14.09	
2010	352	13.08	207	18.11	
2011	405	15.04	191	16.71	
2012	368	13.67	161	14.09	
2013	462	17.16	158	13.82	
2014	593	22.03	83	7.26	
Survival status					<0.0001
Alive	1667	61.92	518	45.31	
All causes of death	1025	38.08	625	54.68	
Prostate cancer (% of total deaths)	717	(70)	444	(71)	
Cardiovascular (% of total deaths)	246	(24)	135	(22)	
Other (% of total deaths)	62	(6)	46	(7)	

SD, standard deviation. * *p*-values were estimated from chi square tests. ** *p*-value was estimated from a *t*-test.

**Table 2:** Risk of all-cause, prostate cancer, and cardiovascular mortality associated with androgen deprivation therapy (ADT) and major cardiovascular comorbidities presented at the baseline in men with metastatic prostate cancer (*N* = 3835).

Variables	All-Cause Mortality		Prostate Cancer Mortality		Cardiovascular Mortality	
	AHR	95% CI	AHR	95% CI	AHR	95% CI
ADT vs. Watchful waiting	0.86 *	(0.78–0.94)	0.86 *	(0.77–0.96)	1.02	(0.83–1.25)
Stroke vs. without	1.05	(0.96–1.16)	0.90	(0.82–1.04)	1.22	(0.99–1.49)
Congestive heart failure vs. without	1.43 *	(1.29–1.60)	1.17 *	(1.02–1.33)	1.59 *	(1.27–1.97)
Coronary Artery diseases vs. without	0.95	(0.85–1.05)	0.92	(0.81–1.03)	0.88	(0.71–1.09)

The adjusted hazard ratio (AHR) was estimated by an inverse probability weighted Cox hazard model. Inversed probability weighting considered age, cancer staging, cancer grade, and year of the cancer diagnosis. Variables included in the Cox model were ADT, stroke, CHF, CAD, and secondary treatment. The Fine and Gray method was adapted to estimate the hazard of PC- and CV-specific survival considering competing risks from other causes of death. * *p* < 0.0001.

**Table 3:** Risk of all-cause, prostate cancer, and cardiovascular mortality associated with androgen deprivation therapy (ADT) in men with metastatic prostate cancer comparing with patients were on watchful waiting (WW) by the status of major cardiovascular diseases presenting at the baseline in age groups of <80 and ≥80 years.

		All-Cause Mortality		Prostate Cancer Mortality		Cardiovascular Mortality	
Status at Baseline	*n* (% of All)	No. of Deaths	ADT vs. WW AHR (95% CI)	No. of Deaths	ADT vs. WW AHR (95% CI)	No. of Deaths	ADT vs. WW AHR (95% CI)
	<80, *N* = 2392						
Stroke ^1^							
Yes							
ADT	457	179	0.86 (0.68–1.08)	126	0.84 (0.65–1.09)	40	1.11 (0.66–1.87)
Watchful waiting	212	115	-	82	-	20	-
No							
ADT	1234	397	0.80 (0.69–0.94) *	307	0.83 (0.70–0.99) *	75	0.95 (0.65–1.37)
Watchful waiting	489	239	-	185	-	45	-
Congestion heart failure ^2^							
Yes							
ADT	257	134	1.03 (0.78–1.35)	87	1.01 (0.73–1.40)	30	1.70 (0.84–3.44)
Watchful waiting	127	77	-	52	-	11	-
No							
ADT	1434	442	0.78 (0.68–0.90) *	346	0.80 (0.68–0.95) *	85	0.87 (0.62–1.22)
Watchful waiting	574	277	-	215	1	54	-
Coronary artery disease ^3^							
Yes							
ADT	64	34	0.91 (0.73–1.15)	23	0.90 (0.69–1.16)	7	1.32 (0.72–2.42)
Watchful waiting	23	10	-	7	-	**	-
No							
ADT	1627	542	0.78 (0.67–0.91) *	410	0.81 (0.68–0.97) *	108	0.90 (0.63–1.28)
Watchful waiting	678	344	-	260	-	64	-
	≥80, *N* = 1443						
Stroke ^1^							
Yes							
ADT	349	171	0.81 (0.64–1.02)	105	0.98 (0.72–1.34)	47	0.81 (0.53–1.23)
Watchful waiting	174	109	-	61	-	34	-
No							
ADT	652	278	0.93 (0.77–1.12)	179	0.83 (0.66–1.03)	84	1.24 (0.86–1.78)
Watchful waiting	268	162	-	116	-	36	-
Congestion heart failure ^2^							
Yes							
ADT	255	140	0.92 (0.71–1.19)	78	0.81 (0.59–1.12)	52	1.40 (0.87–2.27)
Watchful waiting	126	89	-	58	-	23	-
No							
ADT	746	309	0.86 (0.73–1.03)	206	0.91 (0.73–1.13)	79	0.89 (0.63–1.24)
Watchful waiting	316	182	-	119	-	47	-
Coronary artery disease ^3^							
Yes							
ADT	45	17	0.95 (0.73–1.21)	14	0.98 (0.72–1.35)	3	0.85 (0.55–1.32)
Watchful waiting	17	12	-	5	-	6	-
No							
ADT	956	432	0.84 (0.70–1.01)	270	0.84 (0.68–1.05)	128	1.16 (0.82–1.65)
Watchful waiting	425	259	-	172	-	64	-

The adjusted hazard ratio (AHR) was estimated by an inverse probability weighted Cox hazard model. Inverse probability weighting considered age, cancer staging, grade, and year of the cancer diagnosis. ^1^ Variables included in the Cox model were ADT, secondary treatment, congestion heart failure and coronary artery diseases. ^2^ Variables included in the Cox model were ADT, secondary treatment, stroke, and coronary artery diseases. ^3^ Variables included in the Cox model were ADT, secondary treatment, stroke, and congestion heart failure. * *p* < 0.0001. ** Number is under 3. CI: confidence interval.

## Supplementary Materials

The following are available online at https://www.mdpi.com/2072-6694/12/1/189/s1, Table S1: Distribution of baseline patient characteristics according to the status of androgen deprivation therapy (ADT) within the first year of cancer diagnosis by age group. Figure S1: Q-Q plot for age at diagnosis.
