# Supplementary material for: Influence of Baseline Cardiovascular Comorbidities on Mortality after Androgen Deprivation Therapy for Metastatic Prostate Cancer
Source: Cancers (Basel). 2020 Jan 12;12(1):189. doi: 10.3390/cancers12010189 (PMC7016583; doi:10.3390/cancers12010189)
Supplement: Supplementary file 1 [file cancers-12-00189-s001.pdf]

Supplemental Table Distribution of baseline patient characteristics according to the status of androgen deprivation therapy (ADT) within the first year of cancer diagnosis by age group.

Age group (years)	<80					≥80				
	ADT (*N*=1691)		Watchful waiting (*N*=701)		*p* Value	ADT (*N*=1001)		Watchful waiting (*N*=442)		*p* Value
	n	%	*n*	%		*n*	%	*n*	%	
Cancer stage					<0.0001					<0.0001
N1M0	238	14.1	32	4.6		147	14.7	29	6.6	
Any N M1	1453	85.9	669	95.4		854	85.3	413	93.4	
Cancer grade					<0.0001					<0.0001
Well differentiated	37	2.2	32	4.6		19	1.9	14	3.2	
Moderately differentiated	257	15.2	74	10.6		131	13.1	51	11.5	
Poorly differentiated	1326	78.4	482	68.8		807	80.6	287	64.9	
Unknown	71	4.2	113	16.1		44	4.4	90	20.4	
Major cardiovascular comorbidities										
Stroke	457	27.0	212	30.2	0.1106	349	34.9	174	39.4	0.1011
Congestive heart failure (CHF)	257	15.2	127	18.1	0.0767	255	25.5	126	28.5	0.2284
Coronary artery diseases (CAD)	64	3.8	23	3.3	0.5493	45	4.5	17	3.9	0.575
Other comorbidities										
Diabetes	659	39.0	301	42.9	0.0716	374	37.4	157	35.5	0.5035
Hypertension	1276	75.5	552	78.7	0.0848	826	82.5	358	81.0	0.4874
Secondary treatment										
Chemotherapy	240	14.2	-			68	6.8	-		
Enzalutamide/Abiraterone	11	0.7	-			3	0.3	-		
Combination	75	4.4	-			10	1.0	-		
None	1365	80.7	701	100		920	91.9	442	100	
Survival status					<0.0001					<0.0001
Alive	1115	65.9	347	49.5		552	55.1	171	38.7	
All causes of death	576	34.1	354	50.5		449	44.9	271	61.3	
Prostate cancer (% of total deaths)	433	(75)	267	(75)		284	(63)	177	(65)	
Cardiovascular (% of total deaths)	115	(20)	65	(18)		131	(29)	70	(26)	
Other (% of total deaths)	28	(5)	22	(6)		34	(8)	24	(9)	

p-Values were estimated from chi square tests.

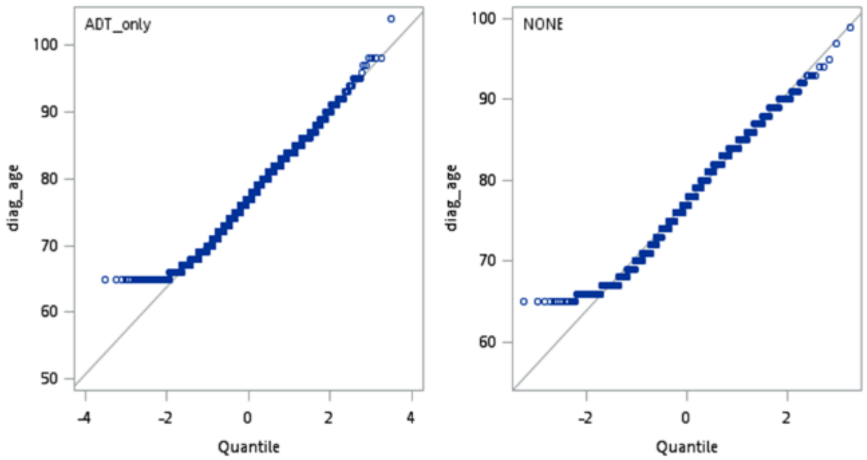

Supplement Figure Q-Q plot for age at diagnosis.
